# Using High-Resolution Ultrasound to Assess Post-Facial Paralysis Synkinesis—Machine Settings and Technical Aspects for Facial Surgeons

**DOI:** 10.3390/diagnostics12071650

**Published:** 2022-07-07

**Authors:** Andreas Kehrer, Marc Ruewe, Natascha Platz Batista da Silva, Daniel Lonic, Paul Immanuel Heidekrueger, Samuel Knoedler, Ernst Michael Jung, Lukas Prantl, Leonard Knoedler

**Affiliations:** 1 Department of Plastic, Hand and Reconstructive Surgery, University Hospital Regensburg, 93053 Regensburg, Germany; marc.ruewe@ukr.de (M.R.); lonic@mclinic.de (D.L.); post@dr.heidekrueger.de (P.I.H.); lukas.prantl@ukr.de (L.P.); leonard.knoedler@stud.uni-regensburg.de (L.K.); 2 Department of Radiology, University Hospital Regensburg, 93053 Regensburg, Germany; natascha.platz-batista-da-silva@ukr.de (N.P.B.d.S.); ernst-michael.jung@ukr.de (E.M.J.); 3 Department of Plastic Surgery and Hand Surgery, Klinikum Rechts der Isar, Technical University of Munich, 81675 Munich, Germany; samuel.knoedler@tum.de

**Keywords:** Depressor Anguli Oris, zygomaticus major, high-resolution ultrasound, facial reanimation, synkinesis, natural smile, facial surgery, facial symmetry, facial palsy, smile restoration

## Abstract

Background: Synkinesis of the facial musculature is a detrimental sequalae in post-paralytic facial palsy (PPFP) patients. Detailed knowledge on the technical requirements and device properties in a high-resolution ultrasound (HRUS) examination is mandatory for a reliable facial muscle assessment in PPFP patients. We therefore aimed to outline the key steps in a HRUS examination and extract an optimized workflow schema. Methods: From December 2020 to April 2021, 20 patients with unilateral synkinesis underwent HRUS. All HRUS examinations were performed by the first author using US devices with linear multifrequency transducers of 4–18 MHz, including a LOGIQ E9 and a LOGIQ S7 XDclear (GE Healthcare; Milwaukee, WI, USA), as well as Philips Affinity 50G (Philips Health Systems; Eindhoven, the Netherlands). Results: Higher-frequency and multifrequency linear probes ≥15 MHz provided superior imaging qualities. The selection of the preset program Small Parts, Breast or Thyroid was linked with a more detailed contrast of the imaging morphology of facial tissue layers. Frequency (Frq) = 15 MHz, Gain (Gn) = 25–35 db, Depth (D) = 1–1.5 cm, and Focus (F) = 0.5 cm enhanced the image quality and assessability. Conclusions: An optimized HRUS examination protocol for quantitative and qualitative facial muscle assessments was proposed.

## 1. Introduction

Facial palsy (FP) patients present with a wide array of muscular, connective, and soft tissue pathologies correlated with different severity levels and comorbidities [1,2,3,4,5,6]. Dysfunctional facial mimic movements constitute a hallmark of FP sequalae and can tremendously impair a patient’s psychological health, social interaction, and overall quality of life [7,8,9,10]. The orchestration of the facial musculature to adequately express a plethora of emotional states is complex and finely balanced [11,12,13]. Research works have shed light on this muscular network in the specific setting of FP [14,15,16,17]. The key muscles for facial symmetry and physiological smile movement have been carved out in micro- and macroanatomical studies [18,19,20,21]. As a protagonist muscle in the perioral region, the Depressor Anguli Oris (DAO) muscle displays a linear origin from the mental tubercle and is inserted on average into the modiolus 10 mm lateral and 10 mm caudal to the oral commissure. Under its muscle tissue, the mental nerve emerges from the mental foramen and connects to the buccal branch (BB) and marginal mandibular branch (MMB) of the facial nerve [21,22,23]. While the BB usually pierces the middle-third of the lateral border of the DAO, the MMB passes the DAO through the lower-third of the lateral border. In PPFP, this phenomenon of DAO dual innervation may be the underlying cause of its hypertonicity in contrast to the weakened Depressor Labii Inferioris (DLI) muscle, which is singly innervated by the MMB [23,24]. Of note, the MMB is commonly represented by one or two branches [25]. The microanatomy of the MMB has been studied by our group previously, demonstrating its axonal load, fascicle structure, and diameter [18,26]. With its function of lowering the corner of the mouth, the DAO is crucial for expressing sorrow and anger, for example [27]. Further, the Zygomaticus Major (ZM) is a cornerstone in the human smile movement [28,29]. The ZM originates at the inferolateral part of the malar eminence in the subzygomatic fossa and runs to the modiolar area [30,31]. Throughout its anatomical course, its appearance shifts from a cylindric shape to a bifid architecture at its insertion locus in 30% of cases [32,33]. This anatomical feature may be the underlying reason for the formation of cheek dimples [28]. The ZM is typically supplied by the facial artery, which pierces its muscular bands in 40% of cases [34]. Raising the corner of the mouth, it is involved in the expression of joy and happiness and thus can be considered, to some extent, a functional opponent to the DAO [28].

The DAO muscle has aroused interest in FP therapy, as its unbalanced and unvoluntary contraction causes a distinctive deformity and divergence between the emotional state and facial expression [27,35,36,37]. DAO dysfunction is indirectly aggravated in FP patients presenting with weakened ZM, since the antagonizing and balancing pull of the ZM is reduced [38,39]. In synkinetic FP patients, these pathognomonic characteristics are particularly prominent [40].

To provide FP patients with adequate and individualized therapy, the diagnostic procedure and preoperative planning tools should be reliable, reproduceable, easy-to-use, widely available, and cost-effective.

The heterogeneous etiology and pathology of FP have counteracted efforts towards general recommendations in FP therapy and diagnostics. For example, the FP therapy guidelines of different associations include varying recommendations regarding the routine diagnostic imaging in new onset FP cases while generally outlining the value of a thorough clinical examination in combination with advanced diagnostic steps [41,42]. However, there is a mounting body of evidence suggesting the beneficial use of high-resolution ultrasound (HRUS) in conjunction with a thorough clinical examination in FP patients, as well as corroborating the overall advancement in visualization quality of a HRUS examination [43,44,45,46,47]. Yet, a step-by-step HRUS examination routine for facial muscle assessment remains to be developed. 

While fulfilling the diverse requirements for clinically applicable diagnostic features, the HRUS examination technique is easy to learn for facial surgeons and may enlarge the diagnostic arsenal. A recent scientific work done by Volk et al. underscored the relevance of device settings for small parts imaging [48,49]. Kehrer et al. proposed defined step-by-step algorithms for US machine settings and the visualization and characterization of microvessels [50,51]. 

The present study aimed to evaluate the technical requirements and different HRUS device properties for facial muscle imaging in PPFP patients showing synkinesis. It was therefore planned to provide a starting guide for facial surgeons less experienced in or even novice at US technology. Based on the results, an optimized HRUS sequence for facial muscle assessment, including the identification of morphologic landmarks and muscular diameter measurement, is proposed. Further, the dynamic evaluation of muscular movement in the DAO region is depicted.

## 2. Materials and Methods

From December 2020 to April 2021, a prospective data acquisition and analysis of US data was conducted on 20 patients seen at the Department of Plastic, Hand, and Reconstructive Surgery at the University Hospital Regensburg, Germany. The inclusion criteria comprised patients featuring a pathologic Sunnybrook Facial Grading System score regarding synkinesis [52]. All HRUS examinations were performed by the first author using US devices with linear multifrequency transducers of 4–18 MHz, including a LOGIQ E9 and a LOGIQ S7 XDclear (GE Healthcare; Milwaukee, WI, USA), as well as Philips Affinity 50G (Philips Health Systems; Eindhoven, the Netherlands). The last author supervised each examination over the entire course and ensured proper prospective data collection. A single-examiner HRUS examination was conducted, providing a consistent and identical workflow in the context of US representing a highly operator-dependent technology [53]. Institutional board review and informed consent were obtained prior to the study. 

### 2.1. High-Resolution Brightness (B)-Mode Examination

B-mode is a basic 2D mode and should lay the groundwork for every US examination. Echo-producing interfaces are scanned by the matrix or linear arrays of transducers in the ultrasound probe. The position of the echo is determined by the time window between the acoustical pulse and its echo, as well as the angular positioning of the transducer. The individual pixel brightness is modulated by the amplitude of the returning US signal. Of note, the typical real-time grayscale picture is formed by the echoes of various acoustic impedances from different tissue layers.

### 2.2. Practical Methodology and Identification of Fundamental Knobology

Useful knobs, switches, and buttons for the assessment of the facial muscle morphology and characteristics in post-facial paralysis synkinesis should be identified and outlined.

### 2.3. Standardized Workflow Protocol

Vital elements of the US exam were disaggregated into key steps for safe facial muscle identification and characterization. A standardized protocol for the US exams was applied.

For the purpose of the present study, the focus was set on the visualization of the DAO muscle morphology, measuring the muscular diameter, and assessing the dynamic behavior in a real-time US examination. The device settings providing specific values for the Frequency (Frq), Gain (Gn), Depth (D), and Focus (F) should be analyzed for an optimized imaging of the facial muscle morphology.

## 3. Results

### 3.1. Technical Requirements

The implementation of a new generation high-resolution US device is recommended. The upcoming generation of US devices accumulates various advancements, including refined reporting features and security measures and improved tissue characterization, as well as novel super-resolution techniques [54]. To this end, US machines should be equipped with high-performance computer chips, typically integrated in the larger pushable-type machines rather than in the portable-type handheld devices.

### 3.2. Practical Methodology

**Figure 1:**
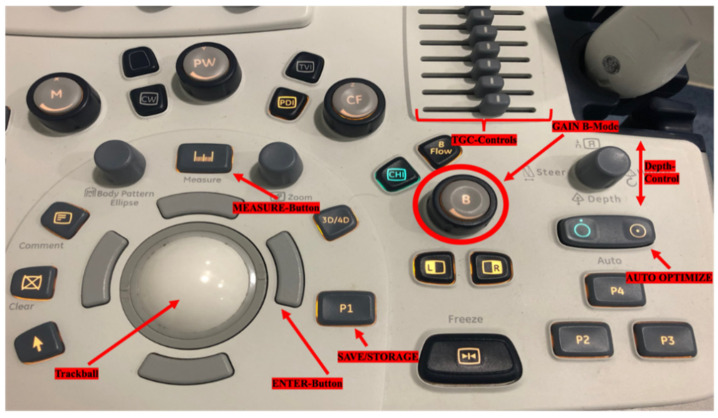
Knobology of a standard ultrasound (US) device. Useful buttons for simplified US usage are marked in red. Time gain controls (TCG) may be all set in the middle position. The depth is varied by the upper and lower lever actions (far right, up). The “Auto Optimize” button harmonizes the image quality. P1 can be programmed to save/store images (in freeze) or cine loops (in a nonfrozen visualization).

Many ultrasound devices share strong similarities of steering panels. The knobology of a standard ultrasound device panel is outlined for the mimetic muscle examiner in Figure 1. Adapted to the specific needs of facial surgeons, useful knobs, switches, and buttons to adjust the parameters are subcategorized into different functional groups in Table 1.

### 3.3. Transducer Selection, Preset Programs, and Device Properties

In this study, higher frequency and multifrequency linear probes ≥15 MHz were helpful adjustments for detecting facial muscles, as well as providing enhanced imaging qualities. Selection of the preset program Small Parts, Breast or Thyroid was linked with a more detailed contrast of the imaging morphology of facial tissue layers in B-mode.

### 3.4. Practical Sequence, Structured Approach, and Standardized Workflow Protocol

**Figure 2:**
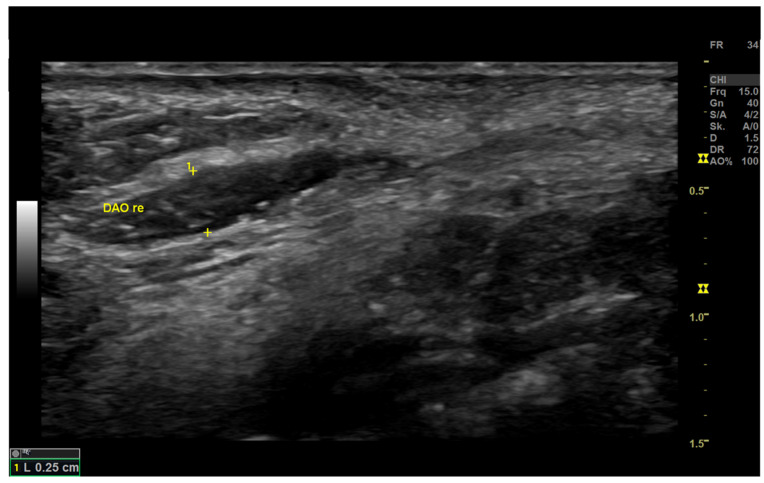
Morphology of the Depressor Anguli Oris (DAO) in the high-resolution brightness (B)-mode examination. Using the preset program Small Parts and the B-mode, the DAO is depicted on the patient’s right facial side. Frq = Frequency [Hz].

In B-mode, the morphology of the skin, subcutaneous, fascia (SMAS), and muscle tissue are depicted (Figure 2). The use of one focus/foci helped to improve the image quality in the targeted area. B-mode images can be optimized with regards to the focus and depth level settings, as well as the preset selection for optimal tissue layer identification. Assessment of the facial tissues is conducted with a superficial high-resolution scan in B-Mode. The centimeter bar located on the right side of the screen helped in the identification and distinction of tissue layers. The dermis and muscle fascia are hyperechoic (bright). In between, fatty components of the subcutaneous and SMAS tissue appear less echoic (darker) in comparison to the dermis.

**Figure 3:**
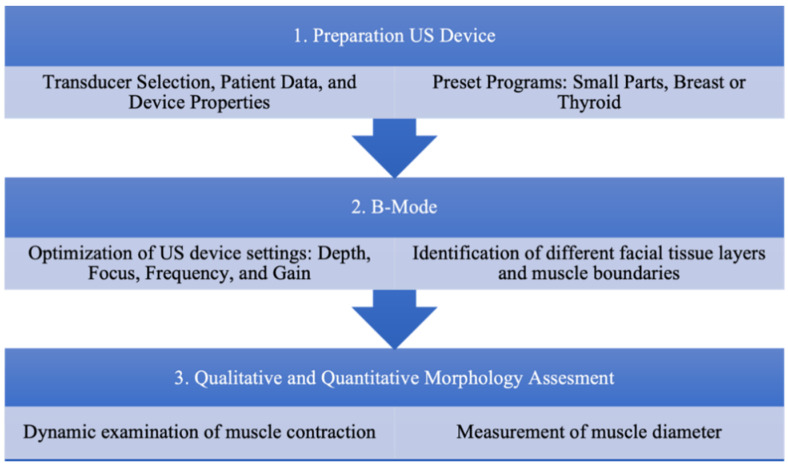
Standardized ultrasound (US) protocol for an effective facial muscle examination. First, the US device is to be prepared for the following examination by selecting an appropriate transducer and preset program. Next step, the brightness (B)-mode can be used to determine different facial tissue layers. Finally, the qualitative and quantitative assessments of muscular morphological features is performed by measuring the cross-sectional muscular diameter and evaluating the muscle functionality in motion.

Key elements of the US examination for efficient and reliable facial muscle identification are summarized in Figure 3. Note that the B-mode should be adjusted and optimized individually for each patient, with an identical sequence for efficiency.

### 3.5. Specific Device Settings

Table 2 provides data of specific US settings for optimized facial muscle assessment.

### 3.6. Cross-Sectional Diameter of Facial Muscles

To assess and objectify post-FP synkinesis, facial muscle cross-sectional diameters can be measured at their midpoint in repose and during maximal contraction. Adjusting the depth and magnification helped to increase the precision.

### 3.7. Practical Sequence and Structured Approach

A structured step-by-step approach decisively shortened the examination times. An examination of related muscles in both facial halves helped to distinguish pathological patterns. Thus, a useful sequence may comprise the following steps:

- Start with using the B-Mode settings and identify the different tissue layers, such as the skin, subcutaneous, fascia (SMAS), and muscle tissue.
- Apply the freeze function and measure the diameter of the facial muscles at their midpoint. Do not forget to store the pictures before unfreezing.
- For a dynamic examination, ask the patient to repetitively perform a broad smile and save the cine loops.

The assessment of dynamic muscle behavior in a real-time US examination using the cine loop function is demonstrated in Video S1.

## 4. Discussion

Facial muscle dysfunction impairs the patient’s quality of life on multiple levels, affecting the oral functionality and mimetic communication [1,3,55]. Thus, effective, reproduceable, and unbiased diagnostic procedures lay the groundwork for introducing targeted therapy concepts in an early post-onset stage. Undoubtedly, the clinical inspection of facial muscles represents a powerful tool in the hands of experienced examiners; yet, it remains subjective. This underscores the necessity to objectify facial muscle malfunction in repose and dynamic movement. HRUS combines wide availability, objective measurements, efficient work- and time flows, noninvasiveness, and cost-effectiveness [56]. Recent advancements in HRUS technology have opened this field for facial surgeons and any specialty involved in the treatment of PPFP cases [50,51,57,58]. To successfully perform an US examination, the facial surgeon and examiner only need a little, yet structured, training, which may be acquired through basic ultrasound courses focusing on the vascular system.

The present work described further how to precisely set ultrasound devices to make them applicable for assessing PPFP and its typical symptoms. It is supposed to address facial surgeons and other disciplines interested in familiarizing themselves with HRUS. Thus, a structured workflow algorithm is proposed to depict and objectify structural (morphological), static, and dynamic facial muscle changes in PPFP. Therefore, to further subdivide the ultrasound technology eligible for facial muscle evaluation, a step-by-step guide providing different steps was now defined in this study. This new structured approach may aid less experienced examiners or even novices in HRUS to gain a clearer view into US technology. Concrete setting values for the Frequency (Frq), Gain (Gn), Focus (F), and Depth (D) for a targeted HRUS examination are provided.

To this end, we used US devices favoring a 15–18-MHz transducer, which is comparable to most contemporary pushable US machine available in almost any larger hospital.

We also underscore that the exact machine settings for facial muscle imaging are indispensable for an efficacious execution. Further, a structured approach using a standardized US mode sequence enhanced the work speed and efficiency in the present study. The authors had previously spent circa 30 min performing HRUS on the face. By applying the proposed sequenced protocol, the examination times could be relevantly reduced to 15–20 min. In addition, US allowed for the efficient measurement of a cross-sectional facial muscle diameter. Visualization of the dynamic facial muscle behavior in PPFP and other types of FP may enhance the examiner’s understanding of the pathological features of muscle morphology. Thus, US may represent a helpful tool in FP diagnostics and may affect the selection of different treatments, such as surgical concepts in the future. Of note, the surgical concepts can be classified into static and dynamic procedures [59,60]. In hyperactive DAO and ZM, dynamic techniques, such as DAO myectomy, selective neurectomy, and Botulinum Toxin A, are valuable therapy options [61]. Rozen et al. showed that the patient achieved significantly enhanced smile excursion following DAO myectomy [62]. DAO muscle transfer as first described by Klebuc et al. may provide symmetrical enhancement in repose and in motion [63,64,65]. The concept reroutes the DAO’s antagonizing downward pull at the modiolus in a smile. This may facilitate the oblique and superior muscle contractility of smile muscles, such as the ZM. Further, the rerouted DAO may enhance the muscular functions of the DLI, which is often found to be dysfunctional in synkinetic patients.

Future studies involving HRUS could investigate facial muscles changes occurring postoperatively, as well as following conservative management. US applying 3D technology or a volumetric assessment may reveal intramuscular changes associated with different (non-)surgical procedures [66]. Further studies are needed to determine how US morphologic and functional findings, such as (facial) muscle size and dynamic muscular behavior, correlate with clinical conditions like muscular hypertonicity. It is the wish of the authors of this paper that, based on the publication of adequate device settings, further US studies are stimulated in the future that contribute to a better understanding of the underlying muscular changes in PPFP.

### Limitations

The results of this study ought to be interpreted in light of the following limitations: The set-up of the US algorithm is based on observational findings in 20 patients with PPFP. While the findings need to be corroborated in larger-scale studies, the sample comprises the most commonly encountered clinical scenarios of PPFP cases. Further, the elucidating research work in this field featured comparable sample sizes [62].

## 5. Conclusions

The proposed US device settings for facial muscle examination may facilitate the facial surgeon’s workflow and result in an enhanced image quality. The structured working protocol may especially help US beginners in conducting more insightful examinations in PFPS patients.

## Figures and Tables

**Table 1:** Functional classification of ultrasound (US) knobology. Group A comprises on-screen options and knobs to store patient data, probe the selection, and classify the ultrasound findings for saved images. Group B includes knobs, switches, and buttons that help to adjust the Contrast, Frequency, Focus, Depth, and other settings. Group C summarizes the functions to quantify distances using a measuring tool.

Knobology for Facial Muscle Assessment with Ultrasound (US)			
**Group A**	Pre-exam buttons	On Display	Patient data button, Probe selection button (usually different linear and convex probes selectable), On-screen buttons for different program presets
	Knobs to classify ultrasound findings for saved pictures	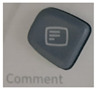	Text editing button
		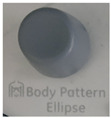	Body pattern ellipse
**Group B**	Adjusting Contrast, Frequency, and Focus	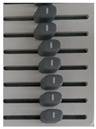	Time Gain Control (TGC) switches
		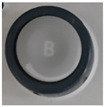	Gain of B-Mode picture
		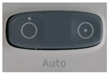	Automated Setting Optimization
		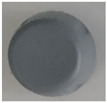	Focus, Frequency
	Basic knobs, switches, and buttons	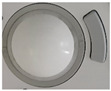	Trackball and Enter-Button
		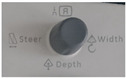	Depth
		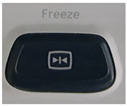	Freeze
		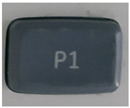	Save Button (fixed images and cine loops, individually programmable)
**Group C**		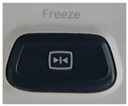	Freeze
	Buttons for measurements	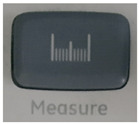	Distance Measurement
		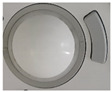	Trackball, Enter

**Table 2:** High-resolution ultrasound (HRUS) settings to examine the functionality and morphology of facial muscles. The following settings allow for the precise assessment of morphological and functional features during a facial muscle examination.

**Facial Muscle Morphology in Ultrasound (US) Examination**	
B-mode (B)	
Probe selection	linear (optimal 15–18 MHz)
Frequency (Frq)	15 MHz
Gain (Gn)	25–35 db
Depth (D)	1–1.5 cm
Focus (F)	0.5 cm

## Data Availability

Not applicable.

## Supplementary Materials

The following supporting information can be downloaded at https://www.mdpi.com/article/10.3390/diagnostics12071650/s1: Video S1. Case example for contrary contraction patterns of nonaffected and synkinetic Depressor Anguli Oris muscles (DAO). The video sequence demonstrates a dynamic HRUS examination comparing DAO morphology in both facial halves while the patient performs a closed-lip smile. On the nonaffected side, the DAO is being stretched while smiling. On the synkinetic side, however, the muscle bulk and cross-sectional diameter of the DAO increase during the smile movement.
